# Families’ Perspectives of Quality of Life in Pediatric Palliative Care Patients

**DOI:** 10.3390/children2010131

**Published:** 2015-03-23

**Authors:** Erin Mary Gaab

**Affiliations:** University of California, 5200 Lake Road, Merced, CA 95343, USA; E-Mail: egaab@ucmerced.edu; Tel.: +1-209-228-4803

**Keywords:** pediatric palliative care, quality of life, children, families, chronic illness, coping, healthcare

## Abstract

Medical and academic institutions began prioritizing Pediatric Palliative Care (PPC) less than two decades ago. Although policies and institutions claim to improve the Quality of Life (QoL) of PPC patients and their families, family-defined QoL remains ambiguous. This research investigates the definitions of QoL for PPC patients according to their primary caregivers. We conducted qualitative, semi-structured focus groups of the primary caregivers of PPC patients. The transcripts were analysed for themes using inductive thematic analysis. Participants included primary caregivers of children currently receiving PPC from a healthcare institution in California. We identified several factors that primary caregivers considered components of QoL for their children. The ability to communicate and adapt or be accepted underpinned the concept of QoL for families. QoL for PPC patients was defined by primary caregivers as being able to communicate in a respectful, controlled, physically- and socially-comfortable environment. Attempts to improve QoL should focus not only on pain and symptom control, but also on enhancing opportunities for children to communicate and maintain a sense of dignity.

## 1. Introduction

### 1.1. Background

Pediatric palliative care (PPC) is a “competent, compassionate, and consistent” mode of healthcare for children with life-limiting and often complex conditions and their families [1]. The first aim of PPC is to improve quality of life (QoL). PPC also serves to minimize suffering, optimize functioning, and meet families’ personal and spiritual needs. It requires the collaboration of health care providers, family members, friends, and patients [2] to assist families over the course of their child’s illness. As medical care evolves technologically and structurally, children receiving PPC are often living well past their prognoses, increasing the resources needed to care for this population.

While the challenges of providing palliative care are increasingly familiar to practitioners of adult medicine, PPC offers a unique set of difficulties. For many reasons, including a lack of awareness, training, reimbursement, and infrastructure, the majority of PPC patients have not been benefiting from palliative care services [3].

The pediatric palliative care movement is in its infancy. Groups including the American Academy of Pediatrics (AAP), the Institute of Medicine, and the World Health Organization began recognizing a need for the improvement of palliative care for children this millennium. George Mark Children’s House, the first free-standing children’s respite and end of life care facility in the USA, admitted their first patient close to one decade ago [4]. California’s Partners for Children (PFC) program, a community-based benefit for the families of children with life-limiting illnesses, was piloted in 2010 [5]. The PFC program allows for children (up to 20 years old) to receive coordinated care, therapies, family education, respite care and pain and symptom management on diagnosis of their life-limiting illness (rather than on a prognosis of six months as the case is with adult palliative care patients). Recently-created Pediatric Palliative Care teams coordinate care, provide families with information needed to make decisions, manage symptoms, make referrals to other professionals [6], and facilitate medical communication [4]. Now it is critical for programs to ensure that they are serving clients’ needs efficiently.

Though policies and services have been developed, research lags behind. Medical and academic institutions only took a strong interest in Pediatric Palliative Care (PPC) about two decades ago [7]. In 2000, AAP called for increased research in the field of pediatric palliative care [3]. Many authors have particularly recognized the need for systematic study of both QoL and quality of care provided at the end of life [8]. Though several measures have been designed to test QoL, this has not been done consistently in PPC populations to our knowledge. However, no QoL scales exist for the PPC population.

To assess one component of the quality of end-of-life care, QoL scales might be employed. HR-QoL (Health Related Quality of Life) and well-being are important measures of psychosocial and other areas of functioning, in addition to physical symptoms. Several tools are available for measuring HR-QoL in generic child populations. These include the PedsQL, CHQ, CHIP and FSII(R) [9]. While a few of these tools have been used widely to assess healthy children and some assess disease-specific groups, they have not been validated in PPC populations. Although PedsQLs that tailor to children’s individual diagnoses might more precisely measure some individual patients’ QoL than generic scales, many PPC patients have more than one illness contributing to their PPC-qualifying status (GMCH personal communication, 2012). Therefore, interpreting the results meaningfully may not be possible for each child’s specific diagnosis. Considering that many PPC patients live with rare conditions, creating individualized definitions of QoL for each PPC-qualifying illness is not feasible or practical.

At present, there are no universal tools to assess the quality of PPC patients’ lives. One of the most widely used scales for assessing healthy children, the PedsQL Generic Core Scale [9] is a 23-item questionnaire designed to measure four multidimensional core scores (physical functioning, emotional functioning, social functioning, school functioning) and three summary scores (total scale score, physical health summary score, psychosocial health summary score). An attempt was made at modifying this tool to assess children receiving PPC. Dr. James Varni drafted such a tool in 2005, entitled the, “PedsQL Comfort Care Module”, by combining elements of the Cancer Module and the Multidimensional Fatigue Scale (Varni, personal communication, 2013). It was specifically designed for use in pediatric hospice and palliative care. However, this tool was not validated or published.

One study has been identified which used a QoL tool to evaluate PPC patients [11]. It incorporated a nonexperimental pretest, posttest design (over a 3-month period) comparing pediatric QoL and family satisfaction. QoL was measured with parent proxy reports of health-related QoL using the PedsQL™ Version 4.0, and family satisfaction was measured with a 31-item self-administered questionnaire designed by project staff [11]. While this study demonstrates the positive effects of a PPC program, the tool may have been flawed and the face validity of the tool has been questioned. Other factors (beyond symptom-control) may need to be considered when measuring QoL in PPC patients. A later study claims that the Pediatric Quality of Life 4.0 contains psychometric properties not valid for measuring HR-QoL within PPC patient populations [12]. To correct this, the evaluators suggest “qualitative interviews with parents whose children have life-limiting illnesses about their perceptions of the items”.

Though much attention has been given to the PedsQL, other and more specific measures exist for assessing children’s QoL. These include the Personal Wellbeing Index [13], KIDSCREEN-52 [14,15], and DISABKIDS [16]. These tools look more broadly at neutral, positive, and negative experiences rather focusing on QoL as the absence of problems. They may, therefore, be more appropriate for pediatric palliative care patients, as their healthcare providers aim to “improve quality of life, minimize suffering, optimize functioning and meet families’ personal and spiritual needs” [1].

The acquisition of information regarding the definition of QoL in PPC patients may direct the improvement of the quality of care these patients receive and demonstrate the need for specific services in this population. This research attempts to define QoL according to primary caregivers who are often used as proxies for their children. The definition of QoL for PPC patients is necessary for the generation of items to include on future QoL measurement tools and may be useful for the development and improvement of organizations which serve PPC patients.

### 1.2. Specific Aims

This research aims to increase knowledge and understanding of QoL in children and adolescents receiving palliative care. Our specific aims are to document families’ definitions of QoL in PPC patients, with emphasis on the following questions: What is QoL for PPC patients according to their primary caregivers? What factors do primary caregivers believe influence QoL and how it changes? How can current scales used to assess QoL be modified to more accurately assess QoL in these patients?

## 2. Methods

### 2.1. Recruitment

Pediatric patients receiving PPC services through two medical institutions in California were identified by staff at these programs. Their parents or primary caregivers were informed of the study in a brochure given to them by staff at their healthcare institution. They were then contacted by a researcher external to their respective institution to be invited to participate. The primary caregivers had children ranging in age from 5–18 years at enrollment. Children receiving PPC included children with expected lifespans limited to childhood. Primary caregivers needed to have an English, Spanish, or Mandarin proficiency level sufficient for comprehension of the PedsQL and KIDSCREEN questions. Primary caregivers with children whose life expectancies were less than four weeks from enrollment in the study were excluded.

### 2.2. Materials

Focus group questions (Box 1) were designed with the help of three multidisciplinary PPC teams at three separate institutions. They were piloted on an initial focus group of primary caregivers two months prior to the commencement of other groups. The questions focused of primary caregivers’ perceptions of QoL for their children. The PedsQL Comfort Care Module Acute Version (Version 3.0) [10], KIDSCREEN-27 [16], and a QoL tool generated by primary caregivers in an initial pilot focus group (Box 2) were handed out to participants in the focus groups.

The focus group questions were piloted on two families receiving PPC. As mentioned, the third QoL scale was drafted in this group and modified with input from PPC providers before the project was initiated at all sites.

Box 1. **Wellbeing / Quality of Life Focus Group Questions.**

1. In two or three minutes, could you please outline for me the background/circumstances of your child’s medical condition that led you to begin receiving pediatric palliative care?
2. In two or three sentences, Could you say a little about what “wellbeing”, “Quality of life” or a life of quality is to you?
3. Please describe how you know your child is having a bad day.
4. Please describe how you know your child is having a good day.
5. How do you know that your child is doing well or poorly …Physically? Socially? Emotionally? Spiritually?
6. Please reflect on the quality of life scales presented [participants shown PedsQL Comfort Care module. KIDSCREEN-25, and a drafted scale]. How would you improve them?
7. Is there anything else you would like to talk about or that you thought about while we were talking?

Box 2 **PPC Wellbeing Questionnaire: How is your child? Please answer the following questions from the perspective of your child. Thinking about the last week (compared to other weeks).**

Expression:1. How well did your child express him-/herself (in sounds, words, coos, moans, sighs, *etc.*)?Not at all

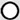
Slightly

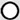
Moderately

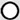
Very

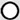
Extremely

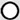
Tension:2. How relaxed was your child?Not at all

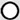
Slightly

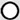
Moderately

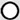
Very

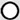
Extremely

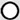
Anxiety:3. How much did the worry or stress in your child’s environment affect your child?Not at all

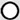
Slightly

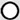
Moderately

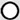
Very

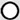
Extremely

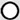
Energy:4. How energetic was your child?Not at all

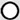
Slightly

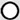
Moderately

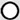
Very

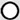
Extremely

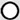
Social:5. How engaged was your child with those around him / her (family, friends, professionals, *etc.*)?Not at all

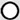
Slightly

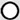
Moderately

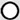
Very

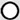
Extremely

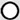
Engagement:6. How engaged was your child with things in his / her environment?Not at all

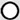
Slightly

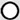
Moderately

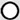
Very

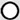
Extremely

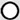
Negative Symptoms:7. How intense have your child’s physical symptoms been (pain, fatigue, sleep issues, nausea, constipation, anxiety, *etc.*)?Not at all

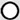
Slightly

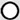
Moderately

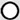
Very

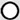
Extremely

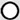
Environment:8. How positively-stimulating has your child’s environment been for him / her?Not at all

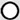
Slightly

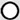
Moderately

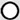
Very

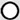
Extremely

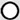

### 2.3. Procedure

Potential participants were identified to the investigator by staff at two PPC-providing institutions in California from lists of current patients. After distributing recruitment brochures to potential participants and receiving oral consent from the participants, the PPC-providers give the Principal Investigator (PI) a list of their phone numbers and emails. The PI called and/or emailed potential participants to invite them to participate in focus groups. The investigator sought written informed consent using standard forms (approved by university and hospital IRBs), documenting the purpose, risks, benefits, and alternatives to participation in the study. Translation services were offered to participants. Consent was solicited at the place most convenient to each participant. Focus groups were conducted and audio recorded by the PI who has five years of experience moderating research and therapeutic focus groups.

Each focus group of three to five participants met for between 54 and 121 min. The participants gathered in locations they specified as convenient to them: about half of the groups in a private room at their healthcare-providing institution and the others met in either a public library or family home. After defining QoL in the context of their children, the primary caregivers were asked to reflect on the PedsQL Comfort Care Module Acute Version (Version 3.0), KIDSCREEN-27 [15], and a QoL tool generated by primary caregivers in an initial pilot focus group.

To maintain participant privacy, no identifying information will be disclosed in this report. After the active phase of data collection, identifiers were removed, leaving only de-identified data for analysis. Raw data was stored in a locked file cabinet at the principal investigator’s office at the University of California, Merced. Data was analyzed on a single password-locked laptop and backed up on a USB stick stored in a locked cabinet.

### 2.4. Analysis

Audio recordings were transcribed by trained, supervised research assistants at UC Merced using an orthographic transcription guide similar to Ordinary Transcription [17]. The transcribed data included the duration of pauses (demarked by one “.” per half-second), crying noises and gestures. The PI and research assistants recorded memos and brief notes throughout the process of data collection and analysis to bracket their perceptions, minimizing biases and compassion fatigue. Having research assistants involved also served to safeguard cultural appropriateness, as the coders came from backgrounds similar to those of the majority of participants (with regard to major ethnicities and socioeconomic statuses). The transcripts were analyzed using Braun and Clarke’s [18] method of inductive thematic analysis. Although the question, “What is QoL for PPC patients?” was the researchers’ primary focus, salient themes which arose spontaneously were also noted. Two research assistants inter-coded each transcript and reviewed themes in order to verify triangulation between the participants and groups.

## 3. Results and Discussion

### 3.1. Participants

Twenty-two participants from two healthcare providing institutions took part in focus groups. Six focus groups were held over an eight-month period in several locations. On a demographics form given to primary caregivers before the initiation of each focus group, the participants identified as being from several ethnicities including Caucasian, African American, Asian and Hispanic. Participants’ children’s illnesses varied (Table 3). PPC patients ranged from 3–18 years old (at the time of the focus groups). The family roles of participants included mothers (adoptive and biological), fathers, and grandmothers. Their household incomes ranged from $10,000 to over $250,000. Their religious beliefs included Christian, Muslim, Jewish, and “none”.

### 3.2. Themes Overview

**Figure 1:**
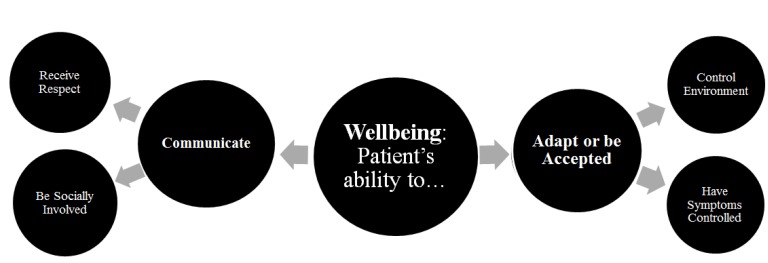
Wellbeing/ Quality of Life components described by primary caregivers of PPC patients.

Primary caregivers spoke about several ways in which they conceptualized “wellbeing” and “quality of life” for their children. Their statements are categorized and described in detail in the themes: *Adapt or be Accepted, Communicate, Control Environment, Receive Respect, Be Socially Involved,* and *Have Symptoms Controlled* (Figure 1).

#### 3.3.1. Adapt or Be Accepted

Families spoke about the need to adapt to their children’s situations in order to maintain wellbeing within their communities. Since PPC patients are rarely treated or conceptualized in the absence of their families, many of parents spoke of accommodations they made. Parents spoke of using “checklists” to help their families adapt. They used faith, social support, and re-framing to help them accept their situation. Their adaptations seemed to allow them to exert more control. Their acceptance of the situation was an expression of maintaining QoL in less controlled environments.

To help his child adapt, one father described using a “checklist” to monitor his child’s condition in a way similar to the way that a healthcare provider might check symptoms:

When she’s unhappy and then you just kinda gotta go through her checklist and (.) okay is she wet (.) is she hungry (.) is it hot (.) is it cold (.) is it bright? …just kind of checklist of what she likes and what she doesn’t like until you figure out what it is that’s upsetting her and then she’ll calm down.

Adapting to the situation required a flexible approach and sometimes an attitude shift. One mother described: “I used to cry every birthday because the kids are in the wheelchairs and they couldn’t do anything y’ know and (..)I started to look at the glass half full instead of half empty more or less and a- it’s- (..) that bit of it’s been a journey”.

When responding to a proposed scale to measure wellbeing, the father suggested the inclusion of positively-focused questions. He stated suggesting that a positive outlook and viewing the child’s condition as “normal” helps his family adapt, enhancing the child’s QoL. Current scales may need to include different components that realistically reflect the abilities of PPC patients:

Some positive stuff: Does your child seem to be engaged um (.) responding positively to stimuli and again this (…) clearly focuses on you know measurable things you can judge that are more within the normal scale so it’s hard to shift for someone who is at the end of the spectrum.

Normalizing the child’s baseline was expressed as a component of QoL by many parents. When situations could not be changed, parents used resources to maintain satisfaction with their child’s condition. A few spoke about using faith and their knowledge about families in similar situations to help them accept their situation. One mother referenced the utility of her spirituality in helping her accept difficult situations:

We always believe that God’s plan and believe that life is a gi-gift … we have to you know do best … for our child (.) and when I came here I see reality about quality of life(.) I worked as a nurse (.) and I see a lot of cases of hospice like hop- hopeless cases… she’s at DNR right now (.) at first I said no? (.) no (.) I can’t (.) whatever I can do I’ll do my best to make her you know the best she could be (.) well right now I accepted the way she is (.) and seeing reality outside that there’s no quality of life then you know do your best but if it’s time for her to go then accept that.

Primary caregivers spoke about accepting and adapting to their children’s situations in order to maintain wellbeing within their families and communities. An open approach to responding to PPC patients’ physical conditions often influenced their QoL, according to their families.

#### 3.3.2. Control Environment

Primary caregivers’ ability to control the environment of PPC patients could be seen as a type of adaptation to their children’s situations. Routines and stable environments were often seen as positive influences on children’s QoL. One mother described her child’s positive response to his consistent schedule:

He seems to really enjoy his daily routine (.) of going on the school bus going to school (.) and then see the day care friends after getting back on the bus and seeing his family in the evening (.) I think just sort of his social routine for the day.

As might be expected, stable environments were perceived to influence wellbeing positively. Both physical and psychosocial components of the environment were mentioned. One mother spoke of her daughter’s reactions to stress at home: “If there’s tension in the house (.) or there’s just arguing or anything like that she’ll respond to it (..) she:: will cry (2.5) she’d have more seizure but she does get a little bit of anxiety.” Children’s environments were mentioned by most parents who participated. One parent even prioritized psychosocial factors before physical symptoms in her definition of QoL for her daughter: “I think her QoL is um being in an emotionally and supportive and positive and happy environment where(.) and she’s physically uh not in pain not suffering.”

Primary caregivers stated that several components of their children’s environments influenced their children’s QoLs. Consistent, calm environments were believed to improve QoL. Another mother spoke of the variability of her son’s physical environment and schedule and its influence on his physical wellbeing:

We’re watching the [baseball] game (.) or we’re kicking back in the hospital room or waiting for food to come and he’s pretty relaxed (.) same day (.) the next day (.) we may have a procedure … there are so many other mitigating factors to what could cause that relaxation or that tenseness.

Being able to control one’s child’s environment was usually seen as positive by the primary caregivers of PPC patients. They hoped to control their children’s wellbeing through adapting to or accepting the factors in their environments.

#### 3.3.3. Have Symptoms Controlled

Children’s physical symptoms were perceived as affecting almost every other aspect of their lives. The necessity of controlling those symptoms was expressed by almost every parent participant. Symptoms mentioned by primary caregivers included pain, motility, seizures, lung or gastroenterological issues, nausea, congestion, fever, fatigue and anxiety.

One mother defined QoL for her child as, “not suffering from a lot of her physical issues that are causing her pain and discomfort.” Another mother spoke of the stress that watching her daughter suffer caused her: “It’s just hard for me as the mother she’s my only child and seeing her suffer every day (..) simple things she cannot go number two or (.) it’s too cold outside and she can’t enjoy that.” As might be expected, physical symptoms played a big part in the QoL of PPC patients, according to their primary caregivers. Another mother contrasted her views of medication use with her son receiving PPC with that of her daughter:

We don’t hold back on medication or anything like that when comes to him (.) with our other two daughters if they don’t absolutely need medication they don’t get it but with him (.) no (.) he takes all sorts of things … we don’t want him to have any pain any suffering in his life (.) I have no tolerance for him to have any pain ’cause he’s had enough already.

The mother prioritized physical comfort over protection from dependence on medication from her son receiving PPC. This contrasted with the reversed prioritization for her well daughters. This type of expression was echoed by a couple of other participants, indicating the perhaps greater importance of physical factors on the QoL of PPC patients.

Obviously, it is necessary for parents to detect physical symptoms in order to adapt to or accept them. One might assume that communicating symptoms would be a bigger issue in nonverbal than verbal children. However, a couple of the verbal children did not communicate physical symptoms with their parents, perhaps in an effort to protect them from the helplessness that they anticipated their parents might feel. One mother spoke of her daughter’s attempts to avoid expressing discomfort:

When her legs hurt (.) she won’t (..) come out and tell me all the time … what she’ll do is “Momma I don’t want to worry you (.) you know you’ve been through so much (.) I don’t want to worry you (.) I don’t want to tell you everything …” but I have been telling her “you have to don’t worry about me I’m here to take care of you” … she’ll just uh sleep all day (.) and then then about a couple of days later she’ll tell me “I was hurting my back was hurting” or you know “I couldn’t walk or good.”

Physical symptoms contribute to the wellbeing of PPC patients, according to all of their primary caregivers. These symptoms require careful monitoring by primary and other caregivers since they may not be directly communicated.

#### 3.3.4. Communicate

Almost all of the primary caregiver participants wanted their children to be “heard” in whatever mode they communicated. Common modes of communication included gestures and sounds, such as “giggling”, “squealing”, “wining”, “fussing”, “crying”, and “cooning”.

The interpretation of these gestures and sounds varied from patient to patient. For example, one mother expressed: “If he squeals he’s happy (.) um he seems calm and at peace.” For others, squealing indicated pain. Smiling was always indicated as a sign of positive wellbeing, although not all children had the ability to smile. One mother described her child’s positive non-verbal communication:

Now she’s happy she’s smiling and she loves to go to school and when I’m getting her ready in the morning she’s happy (.) she’ll giggle or coo …and she’ll smile giggle and coon those are main things (.) it shows her well-being.

Families relied on more than facial expression for interpreting their children’s QoL. One child only communicated when they were experiencing a positive state of wellbeing: “…when he’s well (.) he (.) he does …communicate basically through his facial expressions.” Some parents interpreted symptom-like physical reactions as modes of communication. One patient induced vomit to indicate discomfort:

Mother: if she has cough she’ll vomit (.) she has wet diaper she’ll vomit

P.I.: Do you think that’s connected to her social wellbeing(?)

Father: … this is the only thing she can do to regulate herself (.) so when she would get um (..) you know GI discomfort … so that was her way just to relieve her colicky you know symptoms and so then she’s maintained that- it’s the only thing she can do regulate herself (.) so that’s her response to any discomfort.

As one might expect, physical reactions of discomfort indicated poor QoL to all of the parents interviewed. However, having their children’s expressions heard was also valuable to most parents.

Although participants’ modes of communication varied, all the primary caregivers expressed the importance responding to their children’s utterances. One mother reacted to others’ unwillingness to communicate with her son: “Treat them as a person (.) they’re a member of the family or they’re a member of the community (.) just because they’re different doesn’t mean they can’t communicate (.) you just gotta learn what they’re trying to say.” The importance of others’ responses to PPC patients was mentioned by most participants.

Parents reported that their children tended to communicate more with others on good days (when parents considered them to be in a high state of “wellbeing”). One mother spoke of her infant son’s activeness on good days:

He likes to play little jokes like he: (.) we do his feedings by gravity so you take 60 cc syringe and you attach it to his G-tube … and you pour the formula in (..) well he knows where tha-that G-tube is … he will knock it with his knee so that you’ll get formula all over and he thinks that’s hilarious and I’m like yelling at him for the third time for getting formula all over me [giggles] (..) Oh that’s funny to him (.) that’s really funny.

Although her son’s mischief was not helpful in achieving her aim (feeding him), it was interpreted as playful. Being able to communicate with children and have their gestures and sounds heard was perceived as a component of positive wellbeing to most parents who participated in focus groups. Several parents of PPC patients strove to improve their ability to interact with both family members and people external to PPC patients’ families.

#### 3.3.5. Receive Respect

Primary caregivers spoke of others who treated their PPC patient with dignity and respect. Many of them spoke of the discrimination against, exclusion of, or ignoring of their children. They wanted their children to be listened to and spoken to. Sensitivity to their children’s situations and needs were perceived as factors which contributed to positive states of wellbeing. One mother spoke of an insensitive healthcare provider:

It’s so easy for the doctor to come in … and just like: Stick those things in their mouth and look down her (.) and you’re just like “hey (.) hey (.) ask her could you do that” because when she ask her (.) she’s ready to open her mouth but when you just stick one little thing in there she’s gagging and he’s like (.) “What is goin’ on?” and she’s like “Hey (.) get out of my mouth”

The contribution of respect to their children’s emotional and physical wellbeing was indicated as paramount to several parents. Family members also expressed a need for their situations to be respected. A lack of empathy in the general community was communicated by a father:

I remember when we were first dealing with her … and I remember looking at that and that website and FML and I was like “are you kidding me?” “F--k my life” over these things? [laughter] and I know it’s kind of people complaining over just having fun with it but I was like you have no idea (.) [room laughs] anyway [sighs]

Respect from the general community is a component of QoL to many primary caregivers of PPC patients. According to several of the parents who were interviewed, the sensitivity and respect of those they interacted with played a part in their children’s wellbeing.

#### 3.3.6. Be Socially Involved

In addition to having their children’s communicated needs met, families spoke of their PPC patients as social people requiring social stimulation. Being involved in social activities contributed to their state of wellbeing. Children who were able to communicate about worries, anger, joy and other emotions were perceived as having achieved a high QoL. Two mothers conversed about their young sons’ desires to interact for more than the purpose of meeting physical needs:

Mother 14: He’s Mr. Social (..) he interacts with people with his eyes and he makes vocalizations (..) he (.) giggles (..) smiles at people … there really isn’t anybody he wouldn’t want attention from (.) he’ll just smile at anybody and just (.) if they’re giving him attention (.) that’s his best friend for the moment

Mother 21: …If I think of in terms of [son]’s quality of life um I think that for me its um (..) just being able to allow [son] to (..) be involved and engage in like services and the community as much as my other kids who are non um (.) you know that they are not disable um (.) and just letting him be a part of everything that you know we do as a family I think that’s um (..) pretty important to me [cries]

The parents expressed a preference for others to interact with their children. Their children’s lack of desire for interacting with others was seen as an expression of something being wrong. One mother spoke of her son’s lack of desire to communicate as a sign of decreased wellbeing: “He’s had a god awful day here at school today (.) he’s been fuzzy? (.) he’s been upset? (.) he’s been crying? He doesn’t—he only wants to be held? He doesn’t want to interact? (..) He wants nothing to do with anything”. Social involvement is a component of wellbeing, according to most focus group participants.

## 4. Conclusions

### 4.1. Summary

The abilities of children receiving PPC to adapt to or accept their situations and communicate within them contributed to their QoL, according to their primary caregivers. Their primary caregivers hoped that through symptom-free, controlled, respectful, and accommodating environments, they could maximize their children’s wellbeing. Primary caregivers adapted by normalizing their children’s baselines and focusing on positive parts of their lives. They sought consistent, controllable, calm environments for PPC patients and took measures to minimize their children’s symptoms. Primary caregivers spoke of QoL being enhanced in children who were listened to and treated with respect. According to primary caregivers, “well” children receiving PPC were comfortably communicating in accommodating environments.

### 4.2. Limitations

Unfortunately, as with all qualitative research, knowledge produced from this study may not generalize to other people or settings and quantitative predictions may not be drawn. In order to decrease the biases of the PI and research team, the researchers recorded memos and notes during the processes of interviewing, transcribing and analyzing the data in order to bracket preconceived notions. Complete objectivity is not possible in any research. To minimize bias, debriefing meetings were held after segments of analysis, at which the PI was present.

Despite the limitations of qualitative research, inductive data analysis allows for the generation of new ideas and explanations. Braun and Clarke’s [18] method of inductive thematic analysis was adhered to. Inter-coding and triangulation were also employed to verify the preliminary codes and final themes. This method is ideal for deconstructing participants’ interpretations of constructs, such as “Quality of Life”, and allows for a sensitive approach to generating data. During a couple of the focus groups, tangential topics emerged (such as the need for a medical service in a local hospital), which research participants agreed to work together to obtain, as might occur in Participatory Action Research projects.

### 4.3. Future Directions

The themes herein described might be used to create a tool for use on PPC populations. Through understanding primary caregivers’ definitions of what “a life of quality” is for children receiving PPC, clinicians, caregivers, and others might modify their approach to serving this population. A possible next step could be the creation of a tool for measuring QoL/wellbeing. The modified version of the most appropriate tool(s) might then be used to characterize changes in QoL on initiation of PPC programs or interventions over time.

The diversity of diagnoses, stages of illness, ages, and family perceptions make the development of a tool measuring QoL challenging. However, the same could be said of many generic QoL tools used to assess typically-developing children, another heterogeneous group for which these tools are frequently used.

Another flaw of QoL tools in general is the interdependence of components. For example, children’s ability to communicate may contribute largely to their quality of life when physical symptoms are controlled. However, high levels of symptoms may also impair their ability to communicate, accounting for a greater part of their perceived QoL at a given time. Likewise, the ability to communicate discomfort may also influence children’s abilities to seek treatment for symptoms.

This interdependence of components could also be seen as a flaw of generic QoL scales. Generic scales often begin at a level higher than some PPC patients have ever been able to achieve, such as asking, “Have you been able to run well?” [15].

We hope that the themes generated from the voices of primary caregivers will be used to inform tools, policies and practices involving PPC patients and their families.
